# Intramuscular abdominal hibernoma: case report of a rare tumour and a review of the literature

**DOI:** 10.1093/jscr/rjaa304

**Published:** 2021-02-28

**Authors:** Hooman Baghaie, Erick Chan, Sewwandi Francisco, Haroon Rasheed, Harald Puhalla

**Affiliations:** School of Medicine, Griffith University, Southport, Queensland, Australia; Department of General Surgery, Gold Coast Hospital and Health Service, Southport, Queensland, Australia; School of Medicine, Griffith University, Southport, Queensland, Australia; Department of General Surgery, Gold Coast Hospital and Health Service, Southport, Queensland, Australia; Pathology Queensland, Gold Coast University Hospital, Gold Coast, Australia; School of Medicine, Griffith University, Southport, Queensland, Australia; Department of General Surgery, Gold Coast Hospital and Health Service, Southport, Queensland, Australia; School of Medicine, Griffith University, Southport, Queensland, Australia; Department of General Surgery, Gold Coast Hospital and Health Service, Southport, Queensland, Australia

## Abstract

Hibernoma is a rare benign tumour that was first described by Merkel in 1906. It arises from remnants of brown fat and has a differential diagnosis of lipoma and liposarcoma. This is a case report of a 31-year-old male with a slow-growing mass in the left flank that produced constant pain radiating to the groin. Computerised tomography localised the mass within the external oblique muscle, which showed some heterogeneity and low attenuation. The mass appeared hypodense to muscle on T1 and hyperdense to muscle on T2 weighted magnetic resonance images. Prominent vascularity of the mass was noted. Finally, the lesion was found to be a ‘typical’ hibernoma on core-needle biopsy. It was surgically resected with a cuff of muscle. He recovered without complication, and there is no clinical evidence of recurrence at 6 months.

## INTRODUCTION

Hibernoma is a rare benign tumour of brown adipose tissue that promotes non-shivering thermogenesis. Brown adipose tissue is presented in the foetus and is gradually replaced by white adipose tissue with advancing age. It persists, however, in varying amounts throughout adult life and may be found in the neck, axilla, mediastinum, periaortic and perirenal zones. In the foetus, brown fat has also been identified in the interscapular area, posterior abdominal wall, suprailiac and peripancreatic adipose tissue, and near autonomic ganglia, in addition to the sites described in adults. It is not surprising, therefore, that hibernomas have often been reported to be present in these locations. In this report, we describe a case of hibernoma with unusual site of presentation in the anterior abdominal wall. This has rarely been reported in the English literature. Differential diagnoses for hibernoma include benign lipoma and malignant liposarcoma. Careful investigation and diagnosis are required because of the very different management of the differential diagnoses.

## CASE REPORT

A 31-year-old male was referred to our general surgery team for diagnosis and management of a growing mass in the left flank region. The patient reported incidentally discovering the mass a month ago, and it has since grown and become hard to palpate. He reported a 7/10 sharp constant pain over the flank, which radiates to abdomen and left groin. There were no associated changes to gastrointestinal function or genitourinary symptoms. A systems review was unremarkable. There were no relevant family or social histories. On examination, the abdomen was soft with some tenderness around the firm 5 cm ovoid mass deep in the left lumbar region. The mass was not reducible, and there were no organomegaly or external hernias. The rest of the examination was unremarkable.

**Figure 1:**
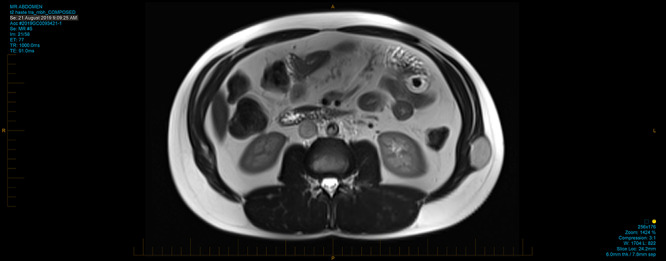
Axial section of abdominal T2 weighted MRI showing hyperintense mass within the left external oblique muscle.

Laboratory studies, including white cell count, haematocrit, electrolytes and liver functions, were all normal. Ultrasound scan of the abdomen showed a 56 × 28 × 27 mm homogenously hyperechoic mass within the muscular abdominal wall with mild internal Doppler vascularity. Provisional diagnosis of fat-containing lumbar hernia was made and a computed tomography (CT) scan of the abdomen was done to confirm the diagnosis. Portal venous phase abdominopelvic CT scan showed an ovoid mass within the external oblique muscle with heterogenous attenuation. There was no calcification, inflammatory changes or evidence of direct invasion into the muscle**.** Appearances were inconsistent with a simple lipoma and magnetic resonance imaging (MRI) was recommended to rule out sarcoma. The abdominal MRI showed a fairly homogenous mass within external oblique muscle with well-circumscribed borders. The lesion was hypodense to muscle on T1 and hyperdense to muscle on T2 weighted images (Fig. 1). There were avid enhancement and some diffusion restriction suggesting high cellularity. Prominent feeder vessels were present. Differentials of undifferentiated sarcoma, fibroma or desmoid tumour were considered. A core biopsy was performed showing brown fat without evidence of atypia consistent with intramuscular hibernoma.

At surgery, a 7-cm linear skin incision was made over the mid-portion of the mass. The mass was localised within the external oblique muscle. It was completely excised with a cuff of muscle by dividing the external oblique muscle. The external oblique muscle, aponeurosis and skin were reapproximated in layers.

**Figure 2:**
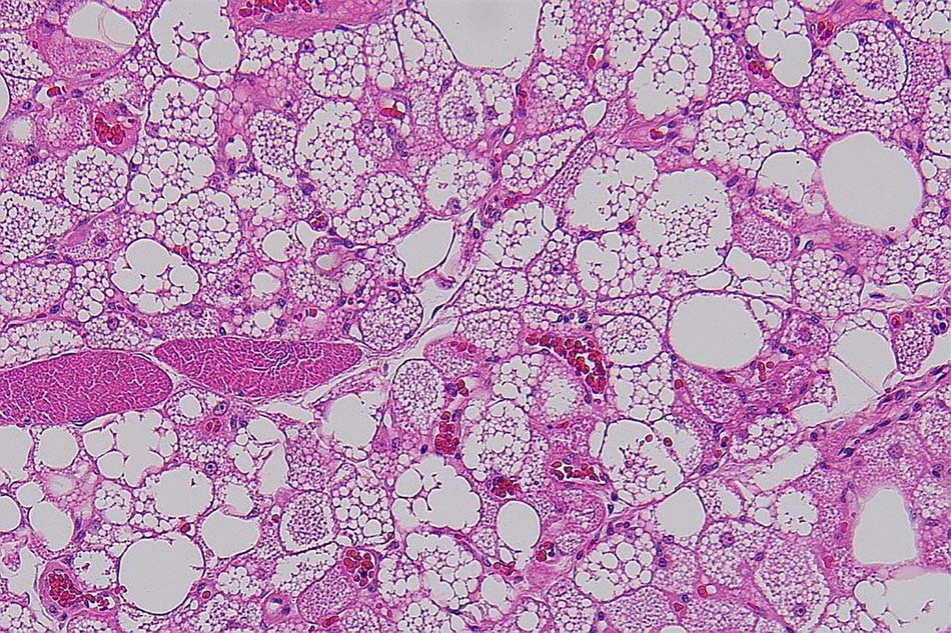
Histophotograph showing lobules of fat with multivaculated brown fat cells without evidence of dysplasia or necrosis.

Macroscopically, it was a fatty mass measuring 48 × 39 × 35 mm, which on cross-section did not reveal any necrosis, haemorrhagic or fibroseptae formation. Microscopically the lesion was composed of lobules of brown far with abundant macrovesicles (Fig. 2). There was no evidence of increased mitotic activity or necrosis. There were no infiltrative borders on the lesions. Fluorescence *in situ* hybridization was used for assessment of *MDM2* gene (12q15) amplification, which was negative. This was performed to rule out any atypical lipomatous tumour.

The postoperative course was uncomplicated. He was reviewed as an outpatient at 2 weeks and again at 6 months. There were no ongoing symptoms or evidence of recurrence.

## DISCUSSION

Hibernomas usually occurs in the second to fourth decade of life and a higher female prevalence [1]. However, a case series [2] of 170 cases found them to be slightly more common in males with a mean age of 38 years. They are slow-growing and present as an asymptomatic mass or with pain associated from pressure on adjacent structures [3]. There has been a paediatric case of supraclavicular hibernoma reported with associated chest discomfort, night sweats, shortness of breath, fatigue and pruritus [4]. The most common anatomic locations included the thigh (30%), shoulder (12%), back (10%), neck (9%), chest (6%), arm (6%) and abdominal cavity/retroperitoneum (5%) [2].

Hibernomas are typically fatty hypervascular lesions that are grossly similar to lipomas. They are well-defined, encapsulated mobile masses. The colour varies from tan to red-brown, depending on the amount of intracellular lipid. While adult adipocytes have an eccentrically placed nucleus within a clear cytoplasm, hibernoma has a central nucleus with a multivacuolated and granular eosinophilic cytoplasm [1].

Four morphologic variants of hibernoma have been identified: typical (82%), myxoid (8%), lipoma like (7%) and spindle cell [2]. The present case belongs to the typical variant as evident by eosinophilic cells multivacuolated. The myxoid variety has a high water content; the lipoma-like variant contains significant amounts of adult fat and commonly affects the thigh. Finally, the spindle cell variant has features of spindle cell lipoma and hibernoma and occurs primarily in the subcutaneous tissues of the neck [5]. There is no size-based classification been established. All the morphologic variants follow a benign course and should be removed completely. The follow-up of 66 cases over a mean period of 7.7 years (range, 6 months and 28 years) showed no recurrence or metastasis, including eight cases of intramuscular tumours [2, 4].

Hibernomas, though rare, should be considered in the differential diagnosis of slow-growing, fibrofatty tumours. Hibernomas and lipomas are difficult to differentiate on imaging. Therefore, histopathological analysis is always necessary for a correct diagnosis. Complete surgical excision is the recommended modality of treatment because of the discomfort that is often associated with the tumour. There is no reported local recurrence, metastasis or any malignant transformation.

## CONFLICT OF INTEREST STATEMENT

None declared.

## FUNDING

None.

## References

[1] Lath N, Familua O, Adu A, Oluwole S. Massive abdominal wall hibernoma: case report and literature review of a rare soft-tissue tumor. J Natl Med Assoc 2011;103:372–4. 21805818 10.1016/s0027-9684(15)30320-5

[2] Furlong MA, Fanburg-Smith JC, Miettinen M. The morphologic spectrum of hibernoma: a clinicopathologic study of 170 cases. Am J Surg Pathol 2001;25:809–14. 11395560 10.1097/00000478-200106000-00014

[3] Carinci F, Carls FP, Pelucchi S, Grandi E, Hassanipour A, Pastore A. Hibernoma of the neck. J Craniofac Surg 2001;12:284–6. 11358103 10.1097/00001665-200105000-00015

[4] Ahmed SA, Schuller I. Pediatric hibernoma: a case review. J Pediat Hematol/Oncol 2008;30:900–1. 10.1097/MPH.0b013e318184e6dd 19131775

[5] Murphey MD, Carroll JF, Flemming DJ, Pope TL, Gannon FH, Kransdorf MJ. From the archives of the AFIP: benign musculoskeletal lipomatous lesions. Radiographics 2004;24:1433–66. 15371618 10.1148/rg.245045120

